# Mr. Davies, on the Doses of Medicines

**Published:** 1800-08

**Authors:** H. Davies

**Affiliations:** Piccadilly


### To the Editors of the Medical and Phyfical Journal.

Gentlemen,

As the profeffed defign of your valuable work, is to conftitute a vehicle of ufeful information in the various departments of phyfic, for the profit and entertainment of your numerous readers; permit me, through the fame channel, to fuggeft a few thoughts, with all due deference, on a fubject which I do not recollect to have been hinted at, in any former number of your mifcellany.

The fubject on which I purpofe to offer a few remarks, is, notwithftanding it may to fome appear trivial, found to be of the utmoft importance in medical practice, viz. *on the neceffity and advantage of gradually augmenting the dose of active remedies in the treatment of obftinate chronic diesases:* An advantage, no doubt, well known to the more fagacious and experienced part of the profeffion, but, perhaps, too much neglected by the raw and injudicious; in confequence of which, I apprehend, that not unfrequently much credit is loft on the part of the practitioner, as well as benefit on that of the patient.

It fcarcely needs to be obferved, that that fenfative organ, the ftomach, through the medium of which the influence of medicines is conveyed to the whole fyftem, lofes, in a great meafure, the ftimulus of the medicine applied, after it has been accuftomed to it for a given time; and hence a reinforcement becomes inevitably neceffary, by an augmentation of the dofe, in proportion as it is capable of receiving it, by which means the curative plan is profecuted with confiderable advantage, and the fymptoms of the difeafe are moft likely to be fubdued.

In no inftance, perhaps, is this obfervation more fully confirmed, than in the treatment of *chlorotic patients*, where a long courfe of fteel, in conjunction with tonic bitters, is indifpenfably neceffary. In this cafe, the fubjects are for the moft part in a ftate of great debility, the ftomach extremely irritable, and ill difpofed, at firft, to retain the naufeating quality of the *ferrum*, in any form in which it can be adminiftered, and in fuch a dofe as is requifite to produce the defired effect. Therefore, by commencing the courfe with fmall dofes, and gradually increafing the quantity during the treatment, we find the ftomach reconciled to it, and the patient feldom complains of naufea or ficknefs; but, on the other hand, derives every benefit, generally fpeaking, by this mode of its adminiftration, which it is expected to communicate to the fyftem.

The fame obfervation will, it is prefumed, hold good, as it refpects respects the administration of other active and important remedies; such as, *hydrargyrum*, *cinchona*, *opium*, *cicuta*, *scilla*, &c. where the cases demand a long continuance of their use. Nor is the hint conveyed, as containing in it any thing new, or not already known, but generally too much neglected; for the writer is well aware of the highly improved state of physic in the present day, and of the numerous practitioners who are justly deemed an honour to the profession. Therefore he flatters himself, that the idea he has suggested will not be construed *by them* as any indirect reflection on their superior skill, but as an humble attempt to serve the general cause, and to promote the good of those, who place an unlimited confidence in, and anxiously expect to receive benefit from, their medical attendants. I am,

GENTLEMEN,

Your's, respectfully,

H. DAVIES.

*Piccadilly,*

*July* 9, 1800.

